# Parity-related molecular signatures and breast cancer subtypes by estrogen receptor status

**DOI:** 10.1186/bcr3689

**Published:** 2014-07-08

**Authors:** Melissa Rotunno, Xuezheng Sun, Jonine Figueroa, Mark E Sherman, Montserrat Garcia-Closas, Paul Meltzer, Tyisha Williams, Sallie Smith Schneider, D Joseph Jerry, Xiaohong R Yang, Melissa A Troester

**Affiliations:** 1 National Cancer Institute, National Institutes of Health, US Department of Health and Human Services, Bethesda, MD 20892, USA; 2 Department of Epidemiology, University of North Carolina, Chapel Hill, NC 27599, USA; 3 Institute for Cancer Research, London SW7 3RP, UK; 4 Department of Biology, Trinity University, San Antonio TX 78212, USA; 5 Department of Veterinary and Animal Science, University of Massachusetts Amherst, Amherst MA 01003, USA; 6 Pioneer Valley Life Sciences Institute, Springfield MA 01199, USA; 7 Genetic Epidemiology Branch, Division of Cancer Epidemiology and Genetics, National Cancer Institute, National Institutes of Health, 9609 Medical Center Drive, RM 6E508, MSC 9769, Bethesda MD 20892-9769, USA

## Abstract

**Introduction:**

Relationships of parity with breast cancer risk are complex. Parity is associated with decreased risk of postmenopausal hormone receptor–positive breast tumors, but may increase risk for basal-like breast cancers and early-onset tumors. Characterizing parity-related gene expression patterns in normal breast and breast tumor tissues may improve understanding of the biological mechanisms underlying this complex pattern of risk.

**Methods:**

We developed a parity signature by analyzing microRNA microarray data from 130 reduction mammoplasty (RM) patients (54 nulliparous and 76 parous). This parity signature, together with published parity signatures, was evaluated in gene expression data from 150 paired tumors and adjacent benign breast tissues from the Polish Breast Cancer Study, both overall and by tumor estrogen receptor (ER) status.

**Results:**

We identified 251 genes significantly upregulated by parity status in RM patients (parous versus nulliparous; false discovery rate = 0.008), including genes in immune, inflammation and wound response pathways. This parity signature was significantly enriched in normal and tumor tissues of parous breast cancer patients, specifically in ER-positive tumors.

**Conclusions:**

Our data corroborate epidemiologic data, suggesting that the etiology and pathogenesis of breast cancers vary by ER status, which may have implications for developing prevention strategies for these tumors.

## Introduction

Parity is associated with lower lifetime breast cancer risk, with changes in differentiation of breast epithelium proposed as a protective mechanism [1]. However, the relationship between parity and breast cancer is complex, and it depends on age, timing of the pregnancy and tumor subtype. Despite decreased lifetime risk, parity has been suggested to increase breast cancer risk transiently following pregnancy [2]. Other analyses suggest a qualitative age interaction, with parity increasing risk early in life and decreasing it in older women [3]. The results of mechanistic studies suggest that increases in hormone levels during pregnancy or in inflammatory microenvironments present during postpartum involution [4,5] may increase breast cancer risk in the years following a birth. However, beyond these temporal trends, there are other nuances in parity–breast cancer associations, with some data suggesting that factors such as age at first birth [2,6], tumor estrogen receptor (ER) status [7-9], menopausal status or age at diagnosis [3] and breastfeeding patterns [10,11] may modify these associations.

Unraveling the complexity of parity and breast cancer risk requires a better understanding of molecular changes associated with parity, together with the recognition of the heterogeneity of breast tumors. In recent studies, some researchers have described parity-induced molecular signatures based on their genome-wide expression profiling of normal breast tissues obtained from healthy women [12-14], which provides new insights into the molecular basis of parity-associated risk protection through alteration of transcription regulation, centrosome organization, RNA splicing, cell-cycle control, adhesion and differentiation. Meanwhile, evaluation of molecular heterogeneity of tumors has led to dramatic changes in our conceptualization of breast cancer, which is now widely recognized as representing at least five molecular subtypes with distinct clinicopathological characteristics and risk factor profiles [9,15-17]. This tumor heterogeneity is particularly important in relation to parity because parity and early age at first full-term birth are associated with reduced risk for ER-positive (ER+) or and (ER + or PR+) breast tumors, but they do not seem to reduce, or they may even increase, lifetime risk for ER-negative (ER-), particularly triple-negative (ER - and PR - and HER2-) breast tumors [8,18-23]. These findings suggest that parity-related factors may influence breast cancer risk through different molecular pathways in ER + and ER - tumors.

In this study, we identified a novel parity-related signature using gene expression profiling data obtained from reduction mammoplasty (RM) specimens. We then tested this parity signature, as well as signatures from previously published studies of normal tissue [12-14,24], in paired tumor and normal tissues collected from breast cancer patients in the Polish Breast Cancer Study (PBCS) [25]. We demonstrate that parity-related molecular processes identified in normal breast tissues of cancer-free women were preserved in normal breast tissues from cancer patients. Furthermore, even ER + tumor tissues persisted in expressing some parity-associated gene expression patterns, suggesting that tumor biology may reflect patient exposures that predate carcinogenesis.

## Methods

### Identification of parity-related gene expression signature

#### Study population and sample handling

Samples were obtained from normal breast tissue of women ages 14 to 70 years who had undergone RM surgery at Baystate Medical Center (Springfield, MA, USA) and whose tissues were banked at the Pioneer Valley Life Sciences Institute (PVLSI) between 2007 and 2010 [26,27]. Specimens were excluded if pathologic assessment of patient-matched, paraffin-embedded tissues demonstrated benign breast disease or neoplastic lesions. Demographic and reproductive information was collected by telephone interview following surgery. The present study was limited to the subjects with complete data on parity, as well as available fresh-frozen tissues so that we could perform gene-profiling analysis, including 54 nulliparous and 76 parous women. The tissue processing, RNA isolation, microarray profiling and preprocessing of gene expression data have been described previously in detail [26,27]. All gene expression data are publicly available through the Gene Expression Omnibus [GSE:16113, GSE:33526].

#### Data analysis

Significance Analysis of Microarrays (SAM) was used to detect differentially expressed genes (with the nulliparous group as the reference) using false discovery rate (FDR < 0.01) to control type I errors due to multiple testing [28]. This method is preferable to fold change–based thresholds methods because it accounts for both variation and multiple testing in gene selection [28]. Genes that were significantly differentially expressed by parity were clustered across all samples using average linkage hierarchical cluster analysis, and results were visualized using Java TreeView software [29]. Ontology classification and pathway analyses were performed on the identified genes using GoMiner software [30].

### Expression of identified parity signature in tumor and paired adjacent normal tissues

#### Study population and sample handling

The PBCS is a population-based case–control study conducted in two major cities in Poland (Warsaw and Łódź) from 2000 to 2003 [25]. The PBCS patients were women ages 20 to 74 years with newly diagnosed, pathologically confirmed *in situ* or invasive breast carcinoma identified using a rapid identification system organized at five participating hospitals and via cancer registries. Fresh tissues from invasive tumors, cancer-adjacent breast tissues and mammary fat tissues were collected from a subset of 227 patients at the time of breast surgery and snap-frozen in liquid nitrogen. Compared to other PBCS patients, the subset of 227 patients had significantly larger and node-positive tumors (Additional file 1: Table S1). A set of 150 paired tumor and cancer-adjacent benign breast samples, which were oversampled for basal-like and luminal cancers and associated tissues, were included in the present study. These samples were selected based on *in vitro* evidence that microenvironments associated with luminal and basal breast cancer subtypes differ [31]. Cancer-adjacent breast tissues used in this study were <2 cm from the tumor edge. Information on clinicopathological, demographic and anthropometric factors was collected from medical records and in-person interviews. Procedures for construction of tumor tissue microarrays, immunohistochemical staining and scoring of key markers, including ER, have been described in detail elsewhere [32,33].

Two-color Agilent 4 × 44K whole-genome arrays (Agilent Technologies, Santa Clara, CA, USA) were used to evaluate gene expression in cancer-adjacent normal tissues. The details regarding tissue processing, RNA isolation, microarray profiling and preprocessing of gene expression data have been described previously [34]. Illumina HumanRef-8 v2 Expression BeadChip arrays (Illumina, San Diego, CA, USA) were used to evaluate gene expression in tumor tissues. In brief, 250 ng of input RNA was amplified and labeled using the Illumina TotalPrep RNA amplification kit (Ambion/Life Technologies, Austin, TX, USA) according to the manufacturer’s recommended protocol. The biotin-labeled cRNAs were quantitated using Quant-iT RiboGreen RNA quantitation reagent (Molecular Probes/Invitrogen, Eugene, OR, USA), and 750 ng was hybridized to Illumina HumanRef-8 v2 Expression BeadChip microarrays (Illumina). Quality checks and data normalization were conducted using standard Illumina preprocessing methods. In particular, the variance stabilization transformation was used, followed by quantile normalization. The number of significant (*P* < 0.05) detection calls was similar across arrays. All gene expression data are publicly available through the Gene Expression Omnibus [GSE: 49175].

#### Data analysis

We used two methods, gene set enrichment analysis (GSEA) [35] and Creighton correlation [36], to test whether the parity signatures from healthy women were associated with parity status in PBCS based on their expression values for the parity-related genes. Both parous and nulliparous women were included in these analyses. GSEA was used to test the enrichment of the parity signature identified in RM patient tissues from PVLSI (PVLSI signature) among parous compared to nulliparous PBCS women. GSEA reveals patterns of gene expression from a given gene set (RM-derived parity signature in this study), even when single-gene analysis reveals very few overlapping, statistically significant genes. A running enrichment score (ES) is calculated by walking down the entire list of probes in the PBCS gene expression array ordered by *t*-test coefficients divided by the standard error values from the parous–nulliparous comparison. This running-sum statistic increases when a given probe is in the PVLSI gene set and decreases otherwise, with the magnitude of increment depending on the strength of the correlation between the probe and the parous–nulliparous comparison in the PBCS data set. We defined the ES as the maximum deviation of the running ES from zero encountered in the random walk. It reflects the degree to which the gene set was overrepresented at the extremes of the entire ranked probe list. Distributions of ES values were created through a permutation procedure and used to calculate the statistical significance of the observed ES values. A permutation-based family-wise error rate (FWER) ≤ 15% was considered as significant. The Creighton correlation method has been described previously [34]. Briefly, standard vectors corresponding to all genes in the parity signature were constructed, with 1 and -1 assigned to genes with fold changes greater and smaller than the median, respectively. A Pearson correlation coefficient was calculated for this standard vector compared to the vector of median centered gene expression for each patient. Patients were classified as positive for parity signature if the Pearson correlation coefficient was greater than zero and as negative if the coefficient was less than zero. Associations of parity signatures with parity status of subjects (nulliparous versus parous) were assessed using unconditional logistic regression by estimating odds ratios (ORs) with 95% confidence intervals (CIs) and by the χ^2^ test (or Fisher’s exact test when expected cell count was less than five) for *P*-values with a threshold for significance equal to 0.05.

The GSEA and Creighton methods are analytically and conceptually different and were used to test two related but distinct questions. We used GSEA to test whether there was a significant subgroup of the genes in the parity signature that was differentially expressed in parous versus nulliparous cancer patients. The Creighton method was used to test whether the parity signature as a whole could predict parity status of cancer patients. Sensitivity analyses were performed to assess heterogeneity of gene expression among cancer patients. GSEAs were stratified by age at first birth (≤ 25 years versus > 25 years prior), interval since most recent birth (< 10 years versus ≥ 10 years) and menopausal status.

### Comparison with previously published parity signatures

We assessed two previously published data sets designed to identify gene expression signatures by parity in normal human tissues: (1) a candidate gene study by Asztalos *et al.*[24], who examined the expression of 64 candidate genes using real-time PCR in mammoplasty benign biopsy specimens from 13 nulliparous and 11 parous age-matched premenopausal women from the University of Illinois at Chicago Hospital (UICH); and (2) a genome-wide expression data set (GeneChip Human Genome U133 Plus 2.0 oligonucleotide array; Affymetrix, Santa Clara, CA, USA) based on normal breast samples from 67 parous and 40 nonparous postmenopausal healthy volunteers recruited at the Sunderby Hospital in Luleå, Sweden (SHL). The SHL expression profiling data set was published in three independent papers (Belitskaya-Lévy *et al.*[12], Russo *et al.*[13] and Peri *et al.*[14]). Because these three research groups used similar statistical methods and generated similar parity signatures, we focused our comparisons on the signature published first, in the paper by Belitskaya-Lévy *et al*. Parity-related genes from the two signatures (UICH and SHL) are listed in Additional file 1: Table S2. The associations between parity and gene expression of UICH and SHL parity signatures were evaluated in normal tissues from the PVLSI study and in tumor and paired cancer-adjacent normal tissues from the PBCS, using the Creighton correlation and GSEA as described above. Data analyses were performed using R (version 3.0.0) [37].

The study protocols were approved by the institutional review boards (IRBs) of each participating institution, and written informed consent was obtained from every participant. The IRB at Baystate Medical Center approved RM sample procurement for the PVLSI women, and the IRB at the University of North Carolina approved the microarrays and statistical analyses of these samples. For the PBCS cases, the collection of breast tissues, questionnaire data and consent for the analyses presented here were covered by the protocol of the Breast, Ovarian and Endometrial Cancer Case–Control Study in Poland, which was approved by the National Cancer Institute Special Studies IRB (OH99CN040).

## Results

### Comparison of sample sets

There were some differences in subject characteristics, source of breast tissues and expression profiling platforms across different studies (Table 1). Although the fresh whole-breast tissue analyzed in PVLSI RMs, PBCS cancer-adjacent normal tissues and SHL core biopsies consisted of both stromal and epithelial tissues, epithelial cells were microdissected in the UICH sample and comprised the dominant cell type in tumors from the PBCS. As is typical for women undergoing RM, PVLSI and UICH patients tended to be more obese and younger (therefore premenopausal) than the SHL and PBCS patients. The PVLSI patients were also younger (mean age = 37 years) and more likely to have had a most recent birth < 10 years prior (68%) than were breast cancer patients in the PBCS group (3%).

**Table 1:** **Differences in study design, experimental approaches and sample characteristics**^**a**^

**Study demographics**	**PVLSI**	**SHL**^**b **^[12]	**UICH**^**b **^[24]	**PBCS cancer-adjacent normal tissue**	**PBCS tumor tissue**
Cancer status	Cancer-free	Cancer-free	Cancer-free	Breast cancer	Breast cancer
Tissue source	Fresh whole tissue	Core needle biopsies	Microdissected FFPE tissue	Fresh whole tissue	Fresh whole tissue
Tissue enrichment	Stroma	Epithelial and stromal	Epithelial	Stroma	Epithelial
Gene expression platform	Agilent 4 × 44K	GeneChip U133Plus 2.0	RT-PCR of 64 genes	Agilent 4 × 44K	Illumina
Samples					
Total	130	107	24	150	
ER+, *n* (%)	N/A	N/A	N/A	117 (78%)	
Parous, *n* (%)	76 (58%)	67 (63%)	11 (46%)	119 (79%)	
Premenopausal, *n* (%)	84 (65%)	0 (0%)	24 (100%)	41 (27%)	
Mean age (±SD), yr	37 (13)	60 (5)	29 (6)	56 (10)	
Mean BMI (±SD)	30^c^ (6)	25 (4)	–	28 (5)	

^a^%, Percentage for categorical variables; BMI, Body mass index; FFPE, Formalin-fixed, paraffin-embedded; N/A, Not applicable; PBCS, Polish Breast Cancer Case–Control Study; PVLSI, Samples obtained from normal breast tissue of 130 women who underwent reduction mammoplasty surgery at Baystate Medical Center in Springfield, MA, USA, and banked at the Pioneer Valley Life Sciences Institute; SD, Standard deviation for continuous variables; SHL, Normal breast samples from 107 postmenopausal healthy volunteers recruited at the Sunderby Hospital in Luleå, Sweden; UICH, Mammoplasty benign biopsy specimens from 24 premenopausal women from the University of Illinois at Chicago Hospital. ^b^From previously published, parity-related gene expression signatures in healthy women [12,24]. ^c^There were 56 (43%) missing BMI values in the PVLSI group.

### Discovery of parity signature in PVLSI mammoplasty specimens

**Figure 1:**
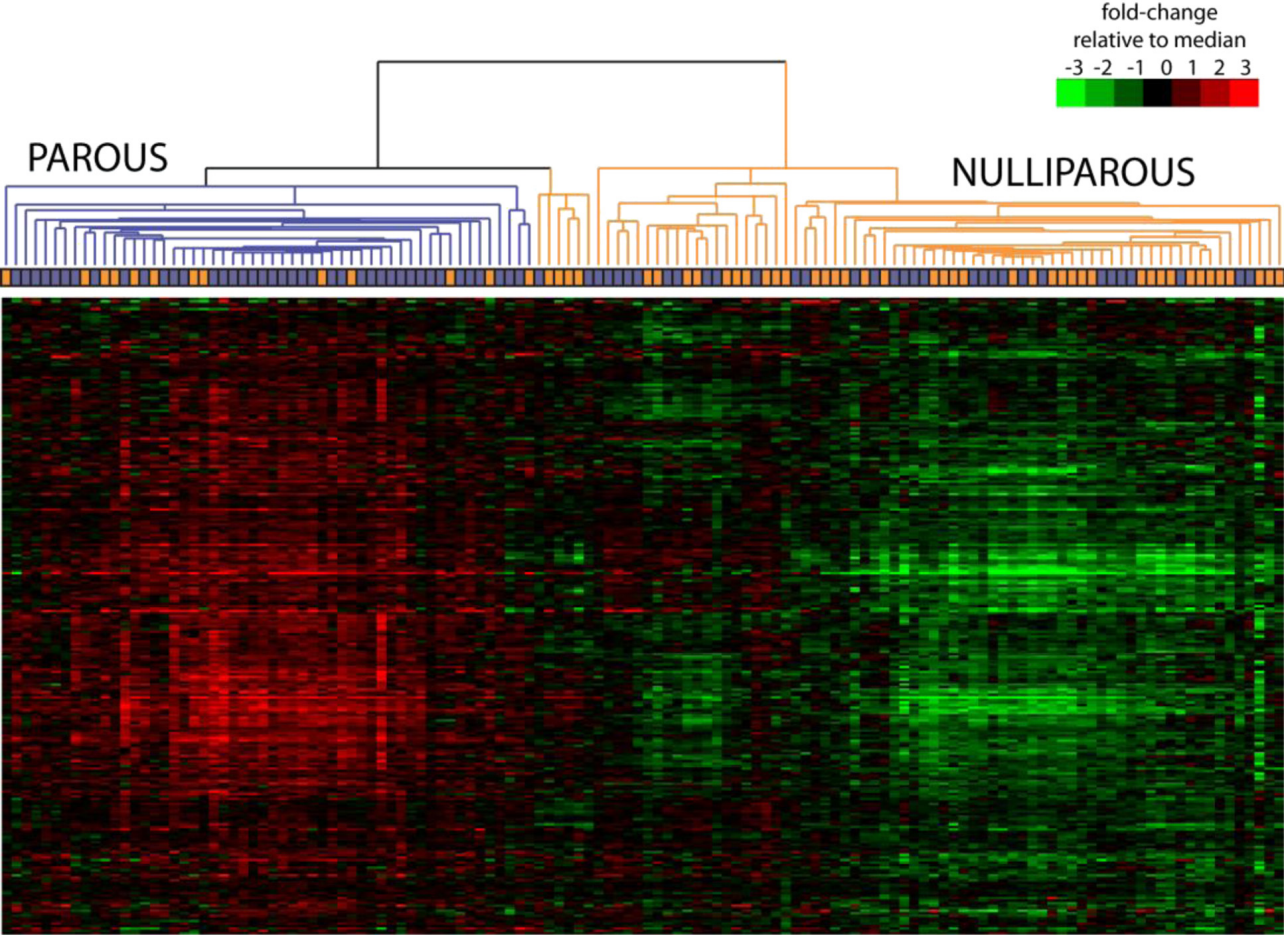
**Unsupervised HeatMap cluster analysis.** The heatmap illustrates our analysis of the 251 parity-related gene expression profiles of normal breast samples from cancer-free women who had undergone reduction mammoplasty surgery at Baystate Medical Center (Springfield, MA, USA) and whose tissue is banked at the Pioneer Valley Life Sciences Institute.

By gene expression profiling analysis of PVLSI samples, we identified 251 upregulated genes (and no downregulated genes) associated with parity (with nulliparous women used as a reference; FDR = 0.008). Gene names, fold changes, FDRs and *P*-values are listed in Additional file 1: Table S3. The prediction of parity status based on the clustering with this parity signature (Figure 1) showed fair agreement with actual phenotype, with greater accuracy seen among parous women (accuracy = 76%) than in nulliparous women (accuracy = 54%). We examined whether the parity-associated gene expression signature varied by age at first birth (≤ 25 years versus > 25 years), interval between most recent birth and tissue collection (< 10 versus ≥ 10 years) and menopausal status in the RM data set. The predictive accuracy of the identified parity signature among parous women did not differ significantly by age at first birth (70% for ≤ 25 years versus 57% for > 25 years; *P* = 0.28), interval since most recent birth (60% for <10 years versus 75% for ≥ 10 years; *P* = 0.19) or menopausal status (62% for premenopausal versus 61% for postmenopausal women; *P* = 0.92).

Pathway analysis suggested that significant genes were associated with immune, inflammation and wound responses. The top ten significant Gene Ontology database biological processes are shown in Table 2. A complete list is available in Additional file 1: Table S4.

**Table 2:** **Top ten Gene Ontology database biological processes enriched by the 251 genes significantly upregulated in normal breast samples of parous compared to nulliparous cancer-free PVLSI patients**^**a**^

**GO category**	**Gene names**	**Total**	**Up**	***P*-value**	**FDR**
[GO: 0002376] Immune system process	KLHL6, HCLS1, LAT2, CD93, SLC7A7, DOK2, NCF4, PTPRC, VNN1, CD38, MAFB, CCL2, ITK, ATP6V0A2, HCK, BLNK, TBX21, FCGR2B, FYB, BPGM, SCIN, CLEC4A, CD244, CD247, TFEB, SNCA, CD48, TNFSF13B, IL6R, TLR3, CDKN2A, P2RX7, SYK, LCP1, C3AR1, ENPP3, ANXA3, VAV1, LCP2, NDFIP1, CD14, TLR1, CD83, CSF1, TLR7, ITGAM, BST1, HLA-DMA, CYBB, EPB42, PRG4, NCF1, WAS, SIX1, TLR6, CCR1, IL1R2, EDA, ITGB2, NCF2, CD28, CCL3	1,040	62	5.3E-19	< 0.001
[GO: 0006955] Immune response	KLHL6, ENPP3, ANXA3, LAT2, LCP2, NDFIP1, NCF4, PTPRC, CD14, VNN1, CD38, TLR1, CD83, CCL2, TLR7, ITK, ATP6V0A2, BLNK, TBX21, FCGR2B, FYB, BST1, CLEC4A, HLA-DMA, CYBB, PRG4, CD247, NCF1, TFEB, SNCA, WAS, TNFSF13B, IL6R, TLR3, TLR6, CCR1, IL1R2, EDA, ITGB2, SYK, P2RX7, NCF2, LCP1, CD28, CCL3, C3AR1	611	46	8.9E-18	< 0.001
[GO: 0006952] Defense response	RNASE6, ANXA3, ADORA3, C1ORF38, ESR2, NDFIP1, PTPRC, CD14, VNN1, NLRC4, TLR1, CD83, CCL2, TLR7, ITK, HCK, BLNK, NOX4, HRH1, CLEC4A, CYBB, CD247, NCF1, SNCA, WAS, CD48, IL6R, LILRB3, TLR3, ALOX5AP, TLR6, AIF1, CCR1, ITGB2, P2RX7, LILRA3, NCF2, CD28, CCL3, C3AR1	578	40	3.1E-14	< 0.001
[GO: 0045321] Leukocyte activation	ANXA3, VAV1, LAT2, CD93, LCP2, NDFIP1, PTPRC, VNN1, TLR1, CD38, CD83, CSF1, TLR7, BLNK, ITGAM, TBX21, HLA-DMA, CD247, SNCA, WAS, CD48, TNFSF13B, TLR3, CDKN2A, TLR6, P2RX7, SYK, LCP1, CD28	336	29	7.5E-13	< 0.001
[GO: 0006954] Inflammatory response	CYBB, ADORA3, C1ORF38, NDFIP1, CD14, IL6R, VNN1, NLRC4, TLR1, TLR3, ALOX5AP, CCL2, TLR7, AIF1, TLR6, CCR1, ITGB2, BLNK, P2RX7, NOX4, HRH1, C3AR1, CCL3	265	23	1.9E-10	< 0.001
[GO: 0002682] Regulation of immune system process	KLHL6, VAV1, LAT2, LCP2, NDFIP1, PTPRC, VNN1, CD38, CD83, MAFB, CSF1, TLR7, ITK, HCK, TBX21, FCGR2B, FYB, SCIN, HLA-DMA, CD247, SNCA, TNFSF13B, WAS, IL6R, TLR3, CDKN2A, TLR6, ITGB2, SYK, P2RX7, CD28, C3AR1	513	32	2.5E-10	< 0.001
[GO: 0002274] Myeloid leukocyte activation	TLR1, TLR3, ANXA3, CSF1, LAT2, CD93, TLR7, TLR6, LCP2, SYK, SNCA, CD48	63	12	6.9E-10	< 0.001
[GO: 0009611] Response to wounding	VAV1, IL10RA, SLC7A7, ADORA3, C1ORF38, LCP2, DOK2, NDFIP1, CD14, GATM, VNN1, TLR1, NLRC4, CCL2, TLR7, BLNK, ITGAM, NOX4, HRH1, CYBB, CD244, WAS, CD48, IL6R, TLR3, ALOX5AP, TLR6, AIF1, CCR1, ITGB2, SYK, P2RX7, LAMP2, PIK3CG, CCL3, C3AR1	671	36	1.1E-09	< 0.001
[GO: 0001775] Cell activation	ANXA3, VAV1, LAT2, CD93, LCP2, NDFIP1, PTPRC, VNN1, CD38, TLR1, CD83, CSF1, TLR7, BLNK, ITGAM, TBX21, HLA-DMA, CD247, SNCA, WAS, CD48, TNFSF13B, TLR3, CDKN2A, TLR6, P2RX7, SYK, LAMP2, LCP1, CD28, PIK3CG	520	31	1.5E-09	< 0.001
[GO: 0050896] Response to stimulus	HCLS1, KLHL6, FPR3, ANG, LAT2, IL10RA, SLC7A7, CACNA2D4, C1ORF38, PTGIS, DOK2, NCF4, PTPRC, VNN1, PTPRO, CD38, NLRC4, FGF1, CCL2, ITK, ATP6V0A2, HCK, PTGS1, BLNK, TBX21, NOX4, HRH1, FCGR2B, FYB, CLEC4A, HTR2B, CD244, CD247, TFEB, SNCA, TNFSF13B, CD48, IL6R, LILRB3, TLR3, ALOX5AP, CDKN2A, AIF1, SYK, P2RX7, LILRA3, LCP1, CRY1, LAMP2, C3AR1, ENPP3, ANXA3, RNASE6, VAV1, ADORA3, LCP2, ESR2, NDFIP1, CD14, GATM, DYNLRB1, TLR1, CD83, TBXAS1, KCNQ1, CYP4V2, C20ORF39, HSPA6, TLR7, CYB5R4, MAOB, ITGAM, ATP6V1F, BST1, HLA-DMA, CYBB, PRG4, NCF1, WAS, ABLIM2, GDF10, TLR6, CCR1, CNGA1, AGPS, IL1R2, EDA, ITGB2, NCF2, CD28, TFEC, PIK3CG, CCL3	3,121	93	2.0E-09	< 0.001

^a^FDR, False discovery rate; GO, Gene Ontology; PVLSI, Samples obtained from normal breast tissue of 130 women who underwent reduction mammoplasty surgery at Baystate Medical Center in Springfield, MA, USA, and banked at the Pioneer Valley Life Sciences Institute; Up, Upregulated.

### Assessment of three parity signatures in mammoplasty samples, cancer-adjacent tissues and cancer tissues

We assessed the enrichment of the three parity signatures (PVLSI, SHL and UICH) in three different tissue sample sets: (1) mammoplasty tissues from PVLSI; (2) cancer-adjacent, histologically normal tissue from PBCS participants with invasive breast cancer; and (3) paired tumor tissues from the same PBCS women. The results of this analysis are shown in Table 3.

**Table 3:** **Associations between parity in PVLSI and PBCS data sets and gene expression enrichment**^**a**^

	**PVLSI signature**		**SHL signature**		**UICH signature**	
PVLSI normal tissues						
GSEA ES^b^	0.98		-0.52		0.71	
GSEA FWER^b^	0% (sig)		N/A		3% (sig)	
Creighton analysis^c^	Negative	Positive	Negative	Positive	Negative	Positive
Nulliparous	37 (69%)	17 (31%)	22 (41%)	32 (59%)	33 (61%)	21 (39%)
Parous	27 (36%)	49 (64%)	45 (59%)	31 (41%)	30 (39%)	46 (61%)
OR (95% CI)^d^	3.95 (1.88 to 8.29)		0.47 (0.23 to 0.96)		2.4 (1.18 to 4.92)	
*P*-value^e^	0.0002 (sig)		N/A		0.015 (sig)	
PBCS cancer-adjacent normal tissues						
GSEA ES^b^	0.52		0.24		0.60	
GSEA FWER^b^	5% (sig)		37%		14% (sig)	
Creighton analysis^c^	Negative	Positive	Negative	Positive	Negative	Positive
Nulliparous	17 (57%)	13 (43%)	16 (53%)	14 (47%)	15 (50%)	15 (50%)
Parous	58 (49%)	61 (51%)	62 (52%)	57 (48%)	62 (52%)	57 (48%)
OR (95% CI)^d^	1.38 (0.61 to 3.08)		1.05 (0.47 to 2.34)		0.92 (0.41 to 2.05)	
*P*-value^e^	0.44		0.90		0.84	
PBCS tumor tissues						
GSEA ES^b^	0.47		0.40		0.58	
GSEA FWER^b^	18%		24%		21%	
Creighton analysis^c^	Negative	Positive	Negative	Positive	Negative	Positive
Nulliparous	16 (53%)	14 (47%)	14 (47%)	16 (53%)	21 (70%)	9 (30%)
Parous	58 (49%)	61 (51%)	55 (46%)	64 (54%)	65 (55%)	54 (45%)
OR (95% CI)^d^	1.20 (0.54 to 2.68)		1.02 (0.56 to 2.27)		1.94 (0.82 to 4.58)	
*P*-value^e^	0.65		0.96		0.13	

^a^CI, Confidence interval; ES, Enrichment score; FDR, False discovery rate; FWER, Family-wise error rate; GSEA, Gene set enrichment analysis; OR, Odds ratio; N/A, Not applicable; PVLSI, Samples obtained from normal breast tissue of 130 women who underwent reduction mammoplasty surgery at Baystate Medical Center in Springfield, MA, USA, and banked at the Pioneer Valley Life Sciences Institute; SHL, Normal breast samples from 107 postmenopausal healthy volunteers recruited at the Sunderby Hospital in Luleå, Sweden; UICH, Mammoplasty benign biopsy specimens from 24 premenopausal women from the University of Illinois at Chicago Hospital. ^b^In GSEA analysis, ES indicates the GSEA enrichment score and FWER is the corresponding family-wise error rate and considered significant (sig) if ≤ 15%. ^c^In Creighton analysis, “Positive” and “Negative” indicate enriched and not enriched expression of parity signature, respectively. ^d^In Creighton analysis, odds ratios (ORs) and 95% confidence intervals (CIs) were obtained by unconditional logistic regression analysis with observed parity as the outcome variable and nulliparity as the reference. ^e^In Creighton analysis, *P*-values were obtained by χ^2^ test or Fisher’s exact test if the expected cell count was less than five, and considered significant (sig) if ≤ 0.05.

Gene expression of the identified PVLSI parity signature was strongly enriched by parity in its PVLSI training set as expected (ES_GSEA_ = 0.98, FWER_GSEA_ = 0%, OR_Creighton_ = 3.95, *P*-value_Creighton_ = 0.0002). Considering all invasive cancer patients (ER + and ER - cases combined), GSEA analyses showed that, though enrichment was weaker than among normal RM tissues, there was a significant enrichment of the PVLSI parity signature in tumor-adjacent normal tissues (ES_GSEA_ = 0.52, FWER_GSEA_ = 5%). The parity signature was further diluted and was then no longer significant in tumor tissues (ES_GSEA_ = 0.47, FWER_GSEA_ = 18%). The results obtained using the Creighton correlation method were consistent with those based on GSEA.

The UICH signature reported by Asztalos *et al*. [24] was significantly positively correlated with parity in normal PVLSI tissues on the basis of both GSEA and Creighton analysis (ES_GSEA_ = 0.71, FWER_GSEA_ = 3%, OR_Creighton_ = 2.4, *P*-value_Creighton_ = 0.01). Similar to our PVLSI signature, the UICH signature also showed a gradient loss of enrichment from normal samples to tumor samples on the basis of GSEA results, but not in the Creighton analysis, which is based on a dichotomous classification.

We did not observe any significant positive association or trend between the SHL signature and parity in the three examined tissue data sets.

### Evaluation of parity signatures in benign cancer adjacent and cancer tissues by estrogen receptor status

To study the distinct effect of parity by breast cancer subtype, we evaluated the enrichment of parity signatures when PBCS samples were stratified by tumor ER status. As shown in Table 4, by GSEA, we observed that our PVLSI signature was significantly enriched by parity in ER + patients (cancer-adjacent normal: ES_GSEA_ = 0.45, FWER_GSEA_ = 12%; tumor: ES_GSEA_ = 0.50, FWER_GSEA_ = 15%), but not among ER - patients (cancer-adjacent normal: ES_GSEA_ = 0.37, FWER_GSEA_ = 21%; tumor: ES_GSEA_ = 0.31, FWER_GSEA_ = 44%). Consistently, the results of the Creighton analysis showed opposite associations of parity with our PVLSI parity signature by tumor ER status in both cancer-adjacent normal tissues (ER+: OR_Creighton_ = 1.53; ER-: OR_Creighton_ = 0.44) and tumor tissues (ER+: OR_Creighton_ = 1.91; ER-: OR_Creighton_ = 0.83), although these associations were not statistically significant. Overall, these trends suggest that the parity signature remains weakly expressed in the tissues of ER + breast cancer patients.

**Table 4:** **Associations between parity and gene expression enrichment of PVLSI parity signature in cancer-adjacent and tumor tissues from PBCS patients by estrogen receptor tumor type**^**a**^

	**Cancer-adjacent normal tissue, ER+**		**Tumor tissue, ER+**		**Cancer-adjacent normal tissue, ER-**		**Tumor tissue, ER-**	
GSEA ES^b^	0.45		0.50		0.37		0.31	
GSEA FWER^b^	12% (sig)		15% (sig)		21%		44%	
Creighton analysis^c^	Negative	Positive	Negative	Positive	Negative	Positive	Negative	Positive
Nulliparous	16 (62%)	10 (38%)	15 (58%)	11 (42%)	1 (25%)	3 (75%)	1 (25%)	3 (75%)
Parous	46 (51%)	44 (49%)	50 (56%)	40 (44%)	12 (43%)	16 (57%)	8 (29%)	20 (71%)
OR (95% CI)^d^	1.53 (0.63 to 3.73)		1.91 (0.45 to 2.6)		0.44 (0.04 to 4.82)		0.83 (0.07 to 9.25)	
*P*-value^e^	0.35		0.85		0.63		1.00	

^a^CI, 95% confidence interval; ER, Estrogen receptor; ES, Enrichment score; FWER, Family-wise error rate; GSEA, Gene set enrichment analysis; OR, Odds ratio; PBCS, Polish Breast Cancer Case–Control Study; PVLSI, Samples obtained from normal breast tissue of 130 women who underwent reduction mammoplasty surgery at Baystate Medical Center in Springfield, MA, USA, and banked at the Pioneer Valley Life Sciences Institute; sig, Significant. ^b^In GSEA analysis, ES indicates the GSEA enrichment score and FWER the corresponding family-wise error rate, with results considered significant (sig) if ≤ 15%. ^c^In Creighton analysis, “Positive” and “Negative” indicate enriched and nonenriched expression of parity signature. ^d^In Creighton analysis, odds ratios (ORs) and 95% confidence intervals (CIs) were obtained by unconditional logistic regression analysis with observed parity as the outcome variable and nulliparity as the reference. ^e^In Creighton analysis, *P*-values were obtained by χ^2^ test or Fisher’s exact test if the expected cell count was less than five, and values were considered significant (sig) if ≤ 0.05.

We also observed differences in parity associations by ER status for SHL and UICH signatures (data not shown); however, these associations were weaker than those for the PVLSI signature.

We refined the parity signature by identifying 41 genes consistently associated with parity across all three GSEA analyses and significantly enriched for PVLSI signature by parity status, that is, in cancer-adjacent normal tissue overall and in ER + as well as ER + tumor tissue analyses (see Table 5 and top 41 genes in Additional file 1: Table S3). A pathway analysis based on these 41 genes revealed the following top 10 biological processes: response to wounding; leukocyte, cell, lymphocyte and T-cell activation; immune system process; cellular response to calcium ion; and immune, inflammatory and defense responses.

**Table 5:** **Forty-one genes associated with parity across all three gene set enrichment analyses**^**a**^

**Gene symbol**	**Gene name**	**FC**^**b**^	***P*-value**^**b**^	**FDR**^**b**^
CD163L1	Scavenger receptor cysteine-rich type 1 protein M160	1.50	0.00006	0
FCGR2A	Fc fragment of IgG, low affinity IIa, receptor for (CD32)	1.61	0.00006	0
ALOX5AP	Arachidonate 5-lipoxygenase-activating protein	1.52	0.00007	0
VNN1	Vanin 1	1.62	0.00007	0
CD33	CD33 antigen (gp67)	1.54	0.00007	0
CD28	CD28 antigen (Tp44)	1.84	0.00007	0
FCGR2B	Fc fragment of IgG, low-affinity IIb, receptor for (CD32)	1.92	0.00009	0
EAF2	ELL-associated factor 2	1.27	0.00009	0
LCP2	Lymphocyte cytosolic protein 2	1.25	0.00011	0
IL10RA	Interleukin 10 receptor, α	1.32	0.00011	0
BLNK	B-cell linker	1.34	0.00012	0
SAMSN1	SAM domain, SH3 domain and nuclear localization, 1	1.43	0.00013	0
CYB5R4	NADPH cytochrome B5 oxidoreductase	1.33	0.00017	0
HTR2B	5-hydroxytryptamine (serotonin) receptor 2B	1.69	0.00031	0
TTC7A	Tetratricopeptide repeat domain 7A	1.17	0.00032	0
ATP6V1F	ATPase, H + transporting, lysosomal 14 kDa, V1 subunit F	1.20	0.00034	0
TLR7	Toll-like receptor 7	1.28	0.00037	0
ANKRD27	Ankyrin repeat domain 27 (VPS9 domain)	1.19	0.00041	0
ITGAM	Integrin, α M	1.55	0.00044	0
CCDC109B	Hypothetical protein FLJ20647	1.27	0.00045	0
CPVL	Carboxypeptidase, vitellogenic-like	1.48	0.00048	0
LEPROTL1	Leptin receptor overlapping transcript-like 1	1.19	0.00062	0.003
CCR1	Chemokine (C-C motif) receptor 1	1.30	0.00068	0.004
S100A10	S100 calcium binding protein A10	1.45	0.00073	0.004
ALG14	Hypothetical protein MGC19780	1.26	0.00093	0.004
SLC7A7	Solute carrier family 7, member 7	1.30	0.00093	0.004
CHRDL1	Chordin-like 1	1.52	0.00095	0.004
S100A4	S100 calcium binding protein A4	1.40	0.00098	0.004
ITGAE	Integrin α E	1.27	0.00098	0.004
CCL2	Chemokine (C-C motif) ligand 2	1.51	0.00102	0.004
CYP4X1	Cytochrome P450, family 4, subfamily X, polypeptide 1	1.49	0.00128	0.004
CCNDBP1	Cyclin D-type binding-protein 1	1.18	0.00144	0.006
TNFSF13B	Tumor necrosis factor (ligand) superfamily, member 13b	1.30	0.00148	0.006
BLVRA	Biliverdin reductase A	1.24	0.00163	0.006
C8orf40	Hypothetical protein BC013035	1.15	0.00174	0.006
BIN2	Bridging integrator 2	1.20	0.00185	0.006
CD48	CD48 antigen (B-cell membrane protein)	1.26	0.00188	0.006
TSG101	Tumor susceptibility gene 101	1.13	0.00199	0.006
GLRX	Glutaredoxin (thioltransferase)	1.40	0.00200	0.006
B3GALT4	β 1,3-galactosyltransferase, polypeptide 4	1.25	0.00206	0.006

^a^FC, Fold change; FDR, False discovery rate; IgG, Immunoglobulin G; PVLSI, Samples obtained from normal breast tissue of 130 women who underwent reduction mammoplasty surgery at Baystate Medical Center in Springfield, MA, USA, and banked at the Pioneer Valley Life Sciences Institute; SAM, Sterile α domain. ^b^Fold change (FC), *P*-value and false discovery rate (FDR) are derived from gene expression comparison of cancer-free parous versus nulliparous PVLSI women. Genes shown were significantly enriched for PVLSI signature by parity status, that is, in analyses of cancer-adjacent normal tissue overall and estrogen receptor–positive and estrogen receptor–positive tumor tissues.

## Discussion

In this study, we identified a significant gene expression signature that was upregulated in breast tissues of parous compared to nulliparous healthy women. Similar to a previously published parity-related signature by Asztalos *et al.*[24] (UICH), which was constructed by selecting genes from the literature, our newly identified signature (selected using an agnostic, supervised analysis) was enriched for inflammation and immune response genes. Although researchers have previously shown in animal studies that upregulation of inflammatory response–related genes was present in the early days of involution and diminished as involution progressed [38-40], our findings suggest that pregnancy has a lasting effect that can be detected many years after completion of a pregnancy. In contrast, the SHL study investigators did not show an increase in immune activity in postmenopausal women and concluded that the upregulation of inflammation and immune response–related genes persists during postpartum involution but wanes after menopause [12]. However, the subjects in the SHL study were much older (mean age = 60 years) than the PVLSI and UICH patients (mean ages = 37 and 29 years, respectively).

We tested three parity-related signatures in genome-wide expression profiling data sets derived from paired tumor-adjacent normal and breast tumor tissues collected from breast cancer cases. We found that parity-related gene signatures were preserved in adjacent normal and tumor tissues, but only among patients with ER + tumors. This is interesting in light of our recent findings suggestive of immune response signatures being dysregulated in normal tissue adjacent to triple-negative breast cancers (P Casbas-Hernandez *et al.*, unpublished data). It is possible that some of the inflammatory genes altered by parity are also responsive to the development of ER - breast cancer and, therefore, that parity-associated signatures become disrupted with progression of ER - disease. The results of our previous work suggest that cancer-adjacent microenvironments show altered expression of wound response genes [27]. ER-, and especially triple-negative, breast cancers may be particularly prone to upregulated cytokines or inflammatory responses, which may lead to the disruption of the parity-associated proinflammatory pathways in ER - breast cancers. However, it is important to note that cytokines that are traditionally associated with inflammation may also play a regulatory role in breast tissue without inducing an immune response [41]. In the absence of direct evidence of inflammatory infiltrates, the inflammation signatures must be interpreted as a change in microenvironment without specifying particular immune suppression or immune avoidance mechanisms. Alternatively, differential expression of parity-associated signatures in ER + versus ER - disease may reflect the differential effect of parity on ER + versus ER - tumors. It appears that, in any case, pregnancy is associated with persistent changes in gene expression that are preserved in women with ER + breast cancer. In a recent study, researchers found that parity decreased the number of hormone receptor–positive luminal cells but had no effect on the basal stem and/or progenitor cells [42]. This suggests that parity may act differently on cells that become luminal versus basal-like breast cancers. If the parity signature is expressed predominantly in normal breast luminal epithelium, then it may be expected that the signature will be expressed only in luminal tumors. Alternatively, the proinflammatory signaling that has been associated with a basal-like stromal response [43] may obscure or disrupt the cytokine expression induced by parity. Identifying the differential effects of parity on distinct cell populations and showing their relevance in human tissues is important for identifying targetable pathways in cancer prevention.

The variations we observed in the expression of the parity signature by ER status are unlikely to have been driven by age, age at first birth or years since most recent birth, because these variables had similar distributions in the ER + and ER - cases evaluated. In addition, the RM-derived parity signature was not significantly associated with tumor characteristics (tumor size, histology, differentiation or lymph node positivity), nor was it associated with overall survival in Kaplan-Meier analyses (data not shown). These data suggest that the signature reflects parity status in ER + breast cancer cases and not a tendency for parous women to have a different prevalence of a particular subtype of breast cancer. However, the weaker association between the gene signature and parity in tumors (relative to normal) is suggestive of the fact that, as tumors progress, they devolve to more unstable states which no longer accurately reflect exposure history.

Comparison of signatures across data sets is challenging because of the differences in profiling platforms, methods of gene selection, patient characteristics (for example, age and body mass index (BMI)) and sample procurement procedures in different studies. Consistent with this, we found limited concordance in parity-related gene content results across different signatures. Some of the previously published signatures [12] were obtained primarily in microdissected epithelium, whereas our novel signature was obtained from the analysis of RM breast tissues that consisted of both epithelium and stroma. The discrepancies in gene signatures emphasize the challenges of obtaining a consistent parity signature with tissues that differ in composition. Our findings add new knowledge about how such signatures persist across the diversity of cell types in breast tissue, an understudied field. Stromal cells predominate in noninvolved, tumor-adjacent tissues, and stromal responses are important to understanding the selective pressures faced by tumors evolving from surrounding stroma [44-47].

In spite of these differences, several signatures showed consistent associations across data sets. Further, some genes were common across signatures. *ESR2* was identified as one of the top significantly upregulated genes in parous women in both the PVLSI and UICH studies. *ESR2* is an ER isoform that has been shown to oppose the proliferative effect of *ESR1*[48]. Increased expression of *ESR2* following pregnancy may reduce the proliferation of breast epithelial cells. Interestingly, in the SHL study, the researchers identified other genes in the ER signaling pathway that were upregulated in parous women, such as *GATA3*. GATA3 is a transcription factor that regulates luminal epithelial cell differentiation in the mammary gland and whose expression is highly associated with ER. Together with the finding of *ESR2* upregulation in the PVLSI and UICH studies, these data suggest that genes involved in the ER-regulated pathways could be under permanent transcriptional modification as a manifestation of a higher degree of parity-associated cell differentiation. In addition, consistent with the SHL study, our parity signature also consisted of upregulated genes. One gene in common between the SHL and PVLSI signatures was TRAF3-interacting, c-Jun N-terminal kinase–activating modulator (*TRAF3IP3*). Genes involved in cell adhesion and differentiation of leukocytes were found to be enriched in both signatures.

Overall, the separation of gene expression by parity status in our data derived from RM patients was not as strong as for other risk factors such as obesity [26], with expression of the signature not found to be in the expected direction in some parous women. This may suggest heterogeneity among patients or the effects of parity on the breast microenvironment being transient. However, other recent findings suggest that parity may alter the composition of breast tissue, notably shifting the tissue composition toward greater adiposity, and these effects may be persistent (X Sun *et al.*, unpublished data). Thus, the persistent inflammatory effects observed in our data may reflect a smoldering inflammatory reaction or a long-lasting shift in stromal composition rather than an acute reaction observed during postlactation involution [5,49]. We cannot exclude some confounding by BMI or age in these analyses, because (1) BMI information was available for only half of the RM patients and (2) most parous women in this data set were also older. Nevertheless, the BMI distributions (mean ± standard deviation (SD)) in parous women (mean = 31, SD = 6) and nulliparous women (mean = 30, SD = 6) were very similar. In addition, BMI is only modestly increased with parity [50], and more substantial changes with BMI are associated with age [51]. We verified that the large majority of the identified parity genes were not part of the age- or BMI-related signature identified in the same population [26,52]. Other variables, such as age at first birth and years since most recent birth, may affect gene expression patterns. However, our present study is not powered to address these questions. None of the subgroup analyses we conducted yielded significant results (FWER > 15%), but future studies addressing these relationships and adequately powered for subanalyses of how plausible confounders (including age, BMI, lactation, number of children, age at first birth and years since most recent birth) influence gene expression–parity associations will be helpful.

## Conclusions

The strength of our study includes the identification of a novel, parity-related signature in stroma-rich breast tissue from healthy women and comparisons with different parity signatures. We tested multiple signatures in both cancer-adjacent and tumor tissues in the same set of breast cancer patients derived from a population-based study to assess whether parity-related signatures were related to tumor progression and subtype heterogeneity. However, our study also has limitations. The small sample size in the training (PVLSI) and test (PBCS) sets prohibited analyses matching on important confounders such as age and BMI or on finer subtypes such as triple-negative or basal-like tumors. In addition, the variation in study subjects, tissues and methods across studies posed challenges to reproducing parity signatures across data sets. Despite these limitations, we found that inflammatory and immune responses and ER regulatory pathways were commonly associated with parity in multiple data sets. Furthermore, parity-related molecular changes appeared to be preserved in breast cancer patients with ER + tumors but disrupted in patients with ER - tumors, which may at least partially account for the observed differential effect that parity has on these two tumor subtypes.

## Abbreviations

BMI: Body mass index; CI: 95% confidence interval; ER: Estrogen receptor; ES: Enrichment score; FDR: False discovery rate; FFPE: Formalin-fixed/paraffin-embedded; FWER: Family-wise error rate; GSEA: Gene set enrichment analysis; OR: Odds ratio; PBCS: Polish Breast Cancer Case–Control Study; PVLSI: Samples obtained from normal breast tissue of 130 women who underwent reduction mammoplasty surgery at Baystate Medical Center in Springfield, MA, USA, and banked at the Pioneer Valley Life Sciences Institute; RM: Reduction mammoplasty; SAM: Significance Analysis of Microarrays; SD: Standard deviation; SHL: Normal breast samples from 107 postmenopausal healthy volunteers recruited at the Sunderby Hospital in Luleå, Sweden; UICH: Mammoplasty benign biopsy specimens from 24 premenopausal women from the University of Illinois at Chicago Hospital.

## Competing interests

The authors declare that they have no competing interests.

## Authors’ contributions

MR, XS, XRY and MAT conceived of and designed the study, performed statistical analyses, interpreted results and drafted the manuscript. JF and MG are the principal investigators of the Polish Breast Cancer Study (PBCS) and contributed substantially to data acquisition and interpretation. MES is a pathologist for PBCS and played a significant role in conducting the pathologic review, scoring key markers and acquiring and interpreting the data. PM carried out gene expression measurements for the PBCS tumor tissues and contributed to data acquisition and experimental analysis. TW, SSS and DJJ are collaborators in the study of samples from the Pioneer Valley Life Sciences Institute (PVLSI) and contributed substantially to the acquisition and interpretation of the data. All authors read and approved the final manuscript, revised it critically for important intellectual content and agreed to be accountable for all aspects of the work to ensure that questions related to the accuracy and integrity of any part of the work are appropriately investigated and resolved.

## Authors’ information

XRY and MAT co–senior authors.

## Supplementary Material

Additional file 1 **Supplementary tables. Table S1.** Distribution of etiologic factors and clinical characteristics among PBCS breast cancer cases with (*n* = 227) and without frozen tumor tissues (*n* = 1,916). **Table S2.** List of genes for the parity signatures previously published by Belitskaya-Lévy *et al.*[12] (202 upregulated and 16 downregulated) and Asztalos *et al.*[24] (8 upregulated and 8 downregulated). **Table S3.** Genes, fold changes, SAM FDRs and *P*-values for the 251 genes significantly upregulated in normal breast samples of parous compared to nulliparous cancer-free PVLSI women. “Yes” in columns “coreN”, “coreN+”, and “coreT+” indicate probes most associated with parity in GSEA analyses based on cancer-adjacent normal tissues overall, cancer-adjacent normal ER-positive tissues, and in ER-positive tumor tissues, respectively. **Table S4.** GO biological processes (*n* = 219) and molecular functions (*n* = 44) significantly enriched (*P* < 0.01) by the 251 genes significantly upregulated in normal breast samples of parous compared to nulliparous cancer-free PVLSI women. Click here for file
